# Efficacy and Tolerability of a Combined Moxifloxacin/Dexamethasone Formulation for Topical Prophylaxis in Phacoemulsification: An Open-Label Single-Arm Clinical Trial

**DOI:** 10.1155/2011/769571

**Published:** 2011-05-25

**Authors:** Cesar Ramon G. Espiritu, Mary Ellen A. Sy, Tommee Lynne G. Tayengco

**Affiliations:** American Eye Center, Level 5, Shangri-La Plaza, EDSA, Corner Shaw Boulevard, Ortigas Center, Mandaluyong City 1554, Philippines

## Abstract

*Background*. The use of a fixed-combination antibiotic corticosteroid for infection prophylaxis in Asian patients undergoing phacoemulsification has not been reported. *Methods*. A 15-day, open-label, single-arm trial of 64 patients for phacoemulsification with intraocular lens (IOL) implantation is described. Patients applied moxifloxacin 0.5%/dexamethasone 0.1% (Vigadexa) eye drops four times daily before and until 15 days after surgery. Anterior chamber (AC) reaction, visual acuity, ocular pain and signs, and intraocular pressure (IOP) were assessed at baseline and on postoperative days 1, 3, 8, and 15. *Results*. At day 15, 55 (91.7%) patients scored 0 (<5 cells) in AC reaction. No surgery-related infection occurred. Mean best-corrected visual acuity improved 0.5 logMAR from baseline to 0.0 logMAR (P < .0001). Mean IOP remained at 12-13 mm Hg over the 15-day treatment. Only 2 patients (3.1%) reported minimum ocular pain. Two (3.1%) patients were shifted to prednisolone acetate for severe inflammation. At the end of the study period, 8.3% were given fluorometholone for 1 week for AC reaction grade >0. No drug-related adverse event was reported. *Conclusion*. Following phacoemulsification and IOL implantation, the topical combination moxifloxacin 0.5%/dexamethasone 0.1% was effective in preventing infection and controlling inflammation and was well tolerated.

## 1. Introduction

Cataract surgery with intraocular lens (IOL) implantation is the most common ophthalmic surgical operation. The mean annual incidence of postoperative endophthalmitis (POE) among cataract patients ranges from 0.05 to 0.14% [1–5]. In practice, ophthalmologists apply topical antibiotic drops to prevent this rare but potentially devastating complication. [3, 6–8]. An American Society of Cataract and Refractive Surgery (ASCRS) survey (*N* = 1312) showed that fourth-generation fluoroquinolones were preferred by most surgeons (81%) for infection prophylaxis after surgery [9]. The topical fourth-generation fluoroquinolone moxifloxacin has proven advantageous over older fluoroquinolones as well as other topically available antimicrobials. It has a broader spectrum of action and excellent penetration into eye tissues and is able to deliver a concentration thousands of times the minimum inhibitory concentration [10–13]. 

Topical corticosteroids such as dexamethasone are applied with infection prophylaxis to minimize, if not eliminate, the inflammatory reaction expected after surgery. It has been reported that treatment with combined steroid-antibiotic eye drops was effective in preventing infection and controlling inflammation after phacoemulsification and IOL implantation [14–16].

In our setting, as well as in other developing nations, financial capabilities of patients and expenditure restrictions from health care organizations demand cost effectiveness. A fixed-combination eye preparation not only helps in cutting costs but also improves patient compliance due to convenience in dosing and application. The combination formulation of moxifloxacin 0.5%/dexamethasone 0.1% (Vigadexa, Alcon Laboratories, Inc., Fort Worth, Tex, USA) is available in the market. Its efficacy and tolerability in ophthalmic surgery has been evaluated [15, 17]. However, its clinical use in Asian patients with cataract has not been reported. The purpose of the present study was to evaluate the efficacy and tolerability of the combination formulation in the prevention of postoperative inflammation and infection following phacoemulsification in predominantly Asian patients.

## 2. Materials and Methods

### 2.1. Patients

This 15-day, open-label, and single-arm clinical trial was conducted at the American Eye Center, Philippines. The center's institutional review board approved the study protocol, which followed the principles set forth in the Declaration of Helsinki. Informed consent was obtained from each patient prior to their participation in the study.

Adult patients (≥18 years) with a diagnosis of cataract underwent clear cornea incision phacoemulsification with IOL implantation. Surgery was performed in only one eye in each patient. Patients who presented with the following conditions were excluded: uncontrolled glaucoma, intraocular hypertension, diabetes mellitus, iris atrophy, chronic or recurrent ocular inflammatory disease (i.e., iritis, scleritis, uveitis, iridocyclitis, and rubeosis iridis), intraocular inflammation, or ocular pain in the study eye prior to the surgery. The use of any ocular antimicrobial drug within 30 days prior to enrollment in the study, nonsteroidal anti-inflammatory drugs or systemic or topical steroids within 14 days prior to enrollment, or a topical prostaglandin analogue four days prior to the surgery until the completion of the study also excluded patients from the study.

### 2.2. Treatment

Patients were instructed to instill one drop from a labeled bottle of moxifloxacin 0.5%/dexamethasone 0.1% (Vigadexa) 4 times a day in the conjunctival sac of the eye to be operated on beginning from day −1 (1 day before the surgery) until day 15 (15 days after the surgery). On day 0 (surgery day), the patient was dosed by the study nurse. A drop was applied prior to and upon completion of the surgery.

The patients underwent phacoemulsification (2.2 mm clear cornea incision) using the Infiniti Vision System (Alcon Laboratories, Inc., Fort Worth, Tex, USA) followed by implantation of a single-piece aspheric hydrophobic acrylic IOL (Acrysof IQ, Alcon Laboratories, Inc., Fort Worth, Tex, USA) in the capsular bag. Viscoelastics used were sodium chondroitin sulfate-sodium hyaluronate (Viscoat, Alcon Laboratories) and sodium hyaluronate (Provisc, Alcon Laboratories) and were removed using coaxial irrigation and aspiration with a vacuum level of 600 mm Hg. All cases were done using the same surgical technique. Preoperative and intraoperative medications included tropicamide/phenylephrine, povidone-iodine local antiseptic and topical proparacaine HCl, and intracameral lidocaine anesthesia.

### 2.3. Patient Evaluation

During the screening visit (within 14 days prior to surgery), baseline values of both eyes were recorded for best-corrected visual acuity (BCVA) in logMAR, and intraocular pressure (IOP) was measured by Goldman applanation tonometry. Patients were examined for the presence of anterior chamber (AC) cells and flare and any pathology of the eyelids, conjunctiva, and cornea through slit-lamp biomicroscopy. A dilated fundus examination was performed to examine the retina, macula, choroid, vitreous, and optic nerve.

Patients were seen postoperatively on days 1, 3, 8, and 15. At each visit, patients were examined for signs of infection, inflammation, and ocular pain. The BCVA and IOP measurements were taken. AC inflammation, a major criterion of effectiveness, was evaluated based on the number of cells per high-power field measured using the narrowest slit beam of the lamp (0.5 at a height of 8 mm) and was recorded on a 0–4 point scale, where 0 indicates less than 5 cells, 1 = 5–10 cells (mild), 2 = 11–20 cells (moderate), 3 = 21–50 cells (marked), and 4 indicates more than 50 cells (hypopyon, severe). Ocular pain was scored by patients subjectively (0 = none, 1 = minimum, 2 = mild, 3 = moderate, 4 = moderately severe, and 5 = severe). Additionally, structural changes and signs of inflammation in the eyelids/conjunctiva and cornea were evaluated by slit lamp (0 = absence of active inflammation and 1 = presence of active inflammation). A dilated fundus examination was performed at exit from the study.

### 2.4. Statistical Analysis

All patients receiving the drug (*N* = 64) were evaluated for safety. Those with at least one follow-up visit after the surgery were included in the per-protocol (PP) analysis (*n* = 60). A Fisher's exact test of independence was employed to evaluate the differences in the percentage of patients with a score of zero for AC cells at each visit before and after treatment. The ocular signs score was analyzed using the Cochran-Mantel-Haenszel (CMH) test with rank score. A Fischer's exact test was applied to evaluate ocular pain. A *t*-test was used to compare IOP change from baseline. Only data from the operated eye were analyzed and reported. The replacement of missing values was adopted for the PP population according to the last value option carried forward technique.

## 3. Results

Sixty-four patients (27 male and 37 female) were enrolled in the study. All were Asians except for two Caucasians. The mean age was 68 years ± 11.4 years (SD) (range from 34 to 86 years). Four patients did not complete the study. Two were lost to followup, and two were shifted to moxifloxacin (Vigamox) and prednisolone acetate (Pred Forte) on day 1 due to severe ocular inflammation.

An increased inflammatory response was observed for the first few days after surgery which gradually declined until day 15 (Table 1). On day 1, 85% of patients had AC cells grade 0–2, while 15% had grade 3. The number of patients with grade 3 AC cells decreased to 1.7% on day 3. On day 15, 91.7% had grade zero AC cells and only 1 patient had moderate inflammation. At the end of the study, 96.9% did not experience eye pain, while 3.1% rated their eye pain as minimum. Signs of active inflammation in the eyelid/conjunctiva and cornea significantly decreased from day 1 postoperatively to day 15. At day 1, inflammation was documented in 9.4% (*n* = 6) of eyes in the eyelid/conjunctiva, while the same was observed in the cornea in 23.4% (*n* = 15) of eyes (Table 2). At the end of the study, signs of inflammation in the eyelid/conjunctiva and in the cornea were seen in only 1 eye and in 2 eyes, respectively. 

**Figure 1:**
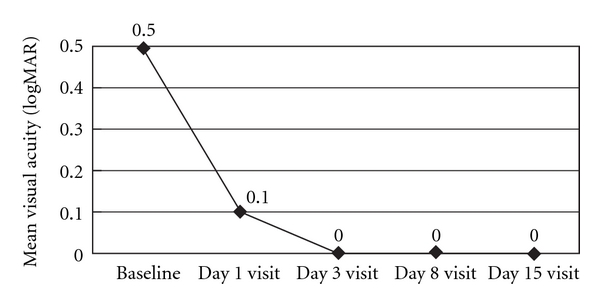
Change in the best-corrected visual acuity of the operative eye before and after treatment.

**Figure 2:**
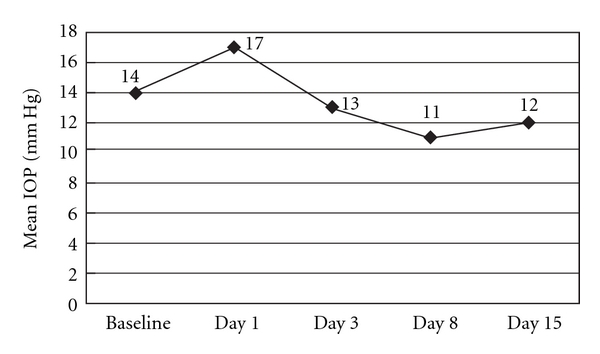
Change in the IOP of the operative eye before and after treatment.

The BCVA improved from a mean of 0.5 logMAR preoperatively to 0.0 logMAR on day 15 (*P* < .0001) (Figure 1). On the day of surgery, the mean IOP was recorded at 17 mm Hg. The mean IOP was maintained postoperatively in the range of 12-13 mm Hg over the course of the 15-day treatment (Figure 2). No abnormality was found in the fundus of the study eyes at exit from the study. No drug-related adverse event was reported.

## 4. Discussion

Our study assessed the efficacy of a fixed-combination moxifloxacin 0.5%/dexamethasone 0.1% formulation (Vigadexa) in the prevention of postoperative inflammation and infection following phacoemulsification in mostly Asian patients. At the completion of the study, a score of 0 for AC cells less than 5 was found in 91.7% (55/60) of eyes. This is comparable to the figure reported in a previous study which was 97% [15]. Patients with an AC reaction higher than grade 0 did not complain of any ocular discomfort or blurring of vision. They were, however, given topical fluorometholone four times daily dosing for one week after discontinuing Vigadexa with resulting resolution of the inflammation. 

On the 1st postoperative day, more than half (55%) had moderate to marked AC cell grading, which decreased to 21.7% by day 3 and to 3.3% by day 8 (Table 1). This is consistent with the postoperative AC reaction pattern the investigators observed in cases where separate antibiotic and corticosteroid eye drops were given after cataract surgery. The postoperative inflammatory pattern in the eyelid/conjunctiva and cornea was judged by the investigators as consistent with previous observations. All these were reflective of the mild ocular changes expected to occur as a result of surgery. 

In this small population of 60 eyes, no surgery-related infection occurred. However, the rarity of the event and the size of the study population did not allow us to make statistically significant conclusions about the effectiveness of the medication in preventing POE. Prior to our single-arm trial, the efficacy of the fixed combination had already been established in a study by Freitas et al. in a Brazilian population [15]. In this randomized, parallel-group trial (*N* = 139), the combination moxifloxacin 0.5%/dexamethasone 1% was as effective in preventing infection and controlling inflammation postoperatively compared to when its individual components were administered concurrently. 

The current study also evaluated the safety and patient acceptance of the fixed-combination preparation of moxifloxacin 0.5% and dexamethasone 0.1%. The formulation was well tolerated by patients; patients did not report any discomfort during or immediately after its application. No corneal or other ocular surface signs attributable to medication toxicity as well as drug-related adverse events were observed during the entire duration of the study. This safety profile mirrors that observed in the study by Freitas et al. [15]. Good patient compliance was determined from patient accounts during follow-up consultations. This can be attributed to the tolerability profile of the drug and ease of administration. Patients will comply with instilling an eye drop that does not sting, burn, cause redness, or blur vision. Furthermore, applying less number of drops makes it easier for patients to remember and adhere to the dosing regimen. With a combination preparation, patients no longer have to wait a minimum of five minutes to instill a drop from a separate medication to prevent a wash-out effect [16, 18, 19]. 

Various fixed-combination preparations have also shown efficacy and safety following cataract surgery. The formulations of netilmicin-dexamethasone, tobramycin-dexamethasone, and neomycin-polymyxin-dexamethasone effectively controlled postoperative inflammation and were well tolerated, as described in comparative trials with Caucasian patients [16, 18, 20].

## 5. Conclusion

The fixed-combination moxifloxacin 0.5%/dexamethasone 0.1% formulation was found to be well tolerated and effective in minimizing inflammation following cataract surgery in Asians. Because Vigadexa is a relatively new medication, further clinical trials with larger number of patients are warranted to further demonstrate and confirm its long-term safety and efficacy profile, particularly in the prevention of endophthalmitis.

## Figures and Tables

**Table 1:** Anterior chamber cells of operative eye, *n* (%).

Anterior chamber cells	Baseline	Day 1	Day 3	Day 8	Day 15
*N*	60	60	60	60	60
0 = Less than 5 cells	60 (100.0%)	2 (3.3%)	26 (43.3%)	49 (81.7%)	55 (91.7%)
1 = Mild: 5–10 cells	0 (0.0%)	25 (41.7%)	21 (35.0%)	9 (15.0%)	4 (6.7%)
2 = Moderate:11–20 cells	0 (0.0%)	24 (40.0%)	1 (20.0%)	2 (3.3%)	1 (1.7%)
3 = Marked: 21–50 cells	0 (0.0%)	9 (15.0%)	1 (1.7%)	0 (0.0%)	0 (0.0%)
4 = Severe: Greater than 50 cells/hypopyon	0 (0.0%)	0 (0.0%)	0 (0.0%)	0 (0.0%)	0 (0.0%)

**Table 2:** Ocular signs of inflammation observed in eyes, *n* (%).

	Baseline	Day 1	Day 3	Day 8	Day 15
Eyelid and conjunctiva					
0	64 (100.0%)	58 (90.6%)	61 (95.3%)	63 (98.4%)	63 (98.4%)
1	0 (0.0%)	6 (9.4%)	3 (4.7%)	1 (1.6%)	1 (1.6%)

Cornea					
0	64 (100.0%)	49 (76.6%)	57 (89.1%)	62 (96.9%)	62 (96.9%)
1	0 (0.0%)	15 (23.4%)	7 (10.9%)	2 (3.1%)	2 (3.1%)

0 = No evidence of active inflammatory signs or significant structural changes or discharge.

1 = Presence of active inflammation signs or significant structural change or discharge.
